# Differences in tourism economic development and its influencing factors among three major city clusters along the middle reaches of the Yangtze River

**DOI:** 10.1371/journal.pone.0299773

**Published:** 2024-05-02

**Authors:** Xiangqiang Li, Ying Huang, Yingying Wang

**Affiliations:** 1 College of Urban and Environmental Sciences, Central China Normal University, Wuhan, Hubei, China; 2 Key Laboratory for Geographical Process Analysis & Simulation of Hubei Province, Wuhan, Hubei, China; 3 Zhongshan Institute, University of Electronic Science and Technology of China, Zhongshan, Guangdong, China; Abdelmalek Essaadi University: Universite Abdelmalek Essaadi, MOROCCO

## Abstract

An in-depth study of the mechanisms governing the generation, evolution, and regulation of differences in tourism economics holds significant value for the rational utilization of tourism resources and the promotion of synergistic tourism economic development. This study utilizes mathematical statistical analysis and GIS spatial analysis to construct a single indicator measure and a comprehensive indicator measure to analyze tourism-related data in the research area from 2004 to 2019. The main factors influencing the spatial and temporal differences in the tourism economy are analyzed using two methods, namely, multiple linear regression and geodetector. The temporal evolution, overall differences and differences within each city group fluctuate downwards, while the differences between groups fluctuate upwards. Domestic tourism economic differences contribute to over 90% of the overall tourism economic differences. Spatial divergence, the proportion of the tourism economy accounted for by spatial differences is obvious, the comprehensive level of the tourism economy can be divided into five levels. The dominant factors in the formation of the pattern of spatial and temporal differences in the tourism economy are the conditions of tourism resources based on class-A tourist attractions and the level of tourism industry and services based on star hotels and travel agencies. This study addresses the regional imbalance of tourism economic development in city clusters and with the intent of promoting balanced and high-quality development of regional tourism economies.

## Introduction

Since the reform and opening-up, tourism has increasingly become an important force in promoting socio-economic progress, so the interrelationship between tourism and tourism and economic development is of increasing interest to scholars [1]. Contemporary regional economics and development economics both reveal the unbalanced law of regional economic development [2]. In the new era, China’s tourism industry is in an important stage of transformation and upgrading, which intensifies the evolution of the tourism economy in space and time. It is very important to analyze the spatial and temporal differences in tourism economic development and optimize the spatial layout of the tourism industry to accelerate the development of tourism in the backward regions, maintain the competitiveness of tourism in developed regions and promote the promotion of tourism integration.

In foreign countries, the issue of tourism economic difference has been studied earlier, and tourism economic difference research has received extensive attention from tourism scholars and geographers. At the beginning of the research, most Western scholars focused on the tourism economy’s drive to local economic development [3,4], recognizing the expanding influence of the tourism economy in the development process. Spatial differences in tourism development affect the coordination of regional development [5], and Western scholars began to focus on the non-equilibrium effects [6,7], the characteristics of spatial and temporal differences [8,9], the evolution of spatial structure [10] and its influencing factors [11, 12] brought about by tourism economic development. Researchers have used correlation coefficients [13], econometrics [14], and model construction [15,16] to explore the inner logic between tourism and economic development. Along with the continuous advancement of China’s tourism economy and the influence of foreign theories and paradigms of tourism economic disparity research, as well as the great driving effect of tourism in reducing regional differences and promoting balanced development, studies on the spatial and temporal variability of China’s tourism economy have been emerging. In terms of research scales, most studies are conducted at the national [17, 18], city cluster [19, 20], and provincial [21] levels, and the spatiotemporal variability of the tourism economy is studied based on multiple scales. For example, Lu Lin et al [17] took 31 provincial units in mainland China as the scope of their study, and analyzed the variation of the tourism economy and its spatial structure characteristics from the perspective of economic geography; in terms of research content, it mainly involved the analysis of tourism economy variability, spatial variation characteristics, tourism economy quality and its influencing factors [22–24], and explored the influencing factors affecting tourism economy spatial and temporal variability from a two-dimensional perspective of space-time, which has a regional balanced development has a catalytic effect. For example, Zhang Shengrui et al [24] analyzed the spatial pattern of development of various types of border tourism in China and its influencing factors from two aspects of spatial variability and spatial autocorrelation, respectively; in terms of research methods, social network analysis, Gini coefficient, Thayer index, spatial autocorrelation analysis, and structural models [22, 25, 26] were widely applied in the measurement of tourism economic differences and the analysis of influencing factors. For example, Sun Xiao et al [19] used the SBM model, Dagum Gini coefficient and decomposition method, and kernel density estimation method to study the regional differences and dynamic evolution of tourism economic growth quality in 36 cities in three northeastern provinces, and Zheng Qunming et al [21] used convergence model to analyze the regional differences of the tourism economy in Hunan province.

In summary, Chinese and foreign scholars have conducted relatively rich research on tourism economic differentiation, spatial and temporal distribution characteristics, structural evolution, and influencing factors, providing a solid foundation for further research on tourism economic differentiation. However, there are still shortcomings in the existing research: Firstly, the research perspective still needs to be constantly expanded, China’s current research based on tourism economic differentiation is more focused on the national, city group, provincial and some specific area perspective, the research related to tourism economic differentiation of different city circles within city groups is relatively rare, so the investigation of tourism economic development differences between other city circles within city groups has practical significance for the coordinated development of the region and theoretical value. Secondly, the research region to be balanced, the current research of domestic scholars in China is mainly concentrated in the eastern coastal region, followed by the western region, the most minor research in the central region, especially the three major city clusters along the middle reaches of the Yangtze River still need to be strengthened, the spatial distribution of tourism economic differences among prefecture-level cities in the three major city clusters along the middle reaches of the Yangtze River has rarely been explored from a spatial perspective. Thirdly, the research indicators are not yet comprehensive, tourism revenue and the number of tourists and other indicators are widely used, few researchers have established a comprehensive system of indicators to assess tourism economic differences. Fourthly, the research methodology needs to be constantly improved, quantitative analysis and qualitative analysis need to be further combined, and the proposed countermeasures need to be more scientific and effective. Fifthly, most of the traditional linear regression, factor analysis, principal component analysis, etc. are used on the influencing factors that cause differences in the tourism economy, while there is a lack of examination of spatial correlation factors, and there is no research that utilizes multiple linear regression and geodetector with dual perspectives yet. Based on this gap, the paper aims to deepen the research perspective, methods, indicators, and analysis of differences. The current situation of tourism economic differences among the three major city clusters along the middle reaches of the Yangtze River will be analyzed in depth from the levels of temporal evolution and spatial differences by means of a single indicator and a comprehensive indicator system, and the main factors of the regional tourism economic differences will be explored from the two perspectives of multivariate linear regression and geographic detector under the consideration of spatial relevance, which will enrich to a certain extent the content and methods of the research on the tourism economic differences of the city clusters both at home and abroad, which will help to provide the basis for the regional tourism planning of the three major city clusters along the middle reaches of the Yangtze River and the realization of the coordinated development of the regional tourism economy, so as to further push forward the construction of the integration of the regional tourism.

## Research design

### Research area

**Fig 1:**
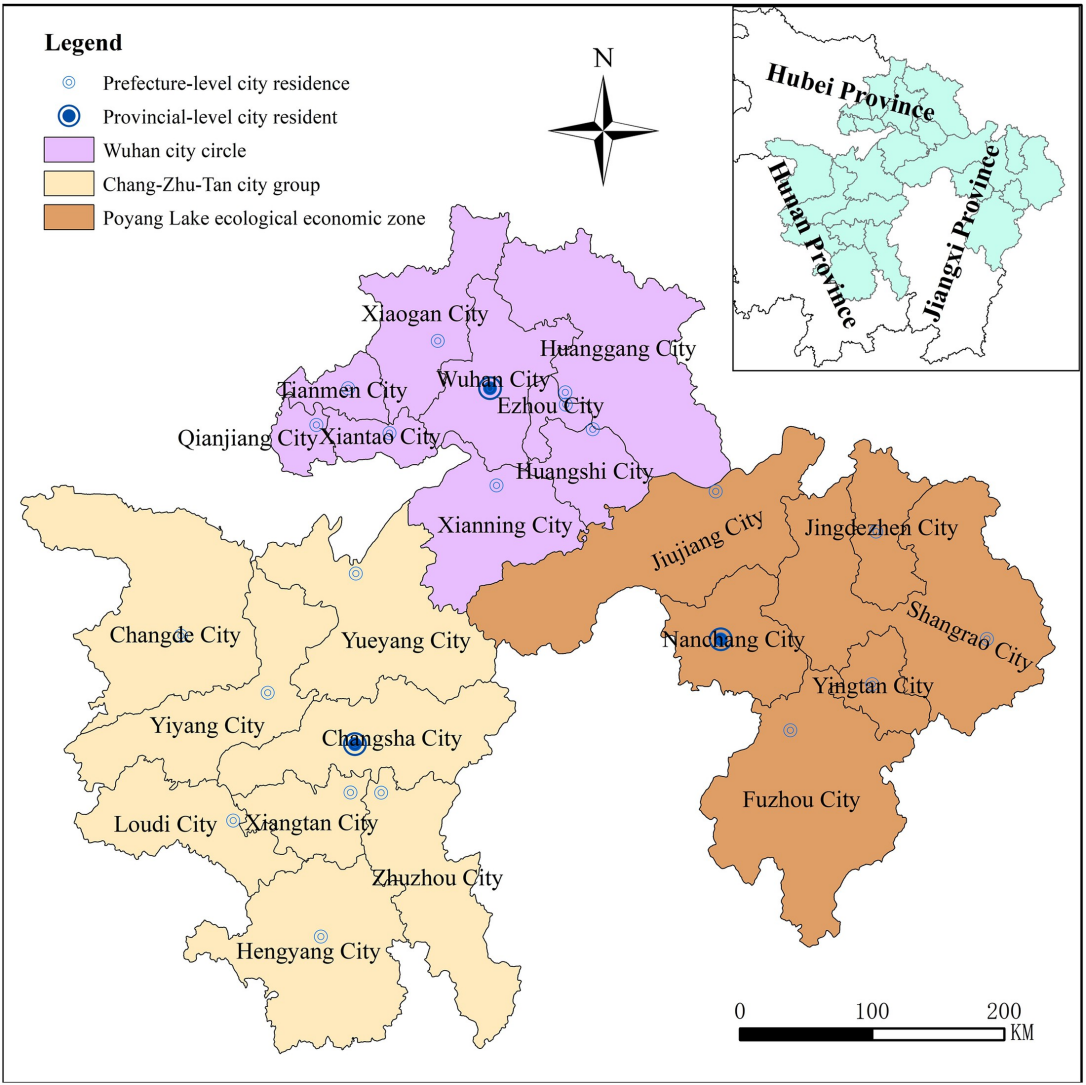
Location and scope of three major city clusters along the middle reaches of the Yangtze River.

The three major city clusters along the middle reaches of the Yangtze River (Fig 1) are uniquely located in the middle of China, bearing east and west, connecting south and north, including 9 cities in Wuhan city circle, 8 cities in Chang-Zhu-Tan city group and 6 cities in Poyang Lake ecological economic zone, which constitute the city clusters along the middle reaches of Yangtze River as the main body, and these three city clusters are in a three-legged spatial posture, and are listed as the national key planning area and the "fourth engine" of China’s economic development. Along with the rapid development of tourism in the three major city clusters along the middle reaches of the Yangtze River, its pillar industry status and related driving role is becoming more and more obvious, while the differences in tourism economy among the cities in the three major city clusters along the middle reaches of Yangtze River are also becoming more and more apparent, and affect the development of tourism economy of the three major city clusters along the middle reaches of Yangtze River as a whole. As an important part of the strategy to support the "fourth pole" of China’s economic development and the rise of central China, tourism cooperation among the three major city clusters along the middle reaches of the Yangtze River has a very important position [27]. An in-depth exploration of the generation, evolution, and regulation mechanism of tourism economic differences are of significant practical value to improve the coordinated development of the tourism economy in the three major city clusters along the middle reaches of the Yangtze River.

### Data sources

Tourism data and socio-economic data of three major city clusters along the middle reaches of the Yangtze River are obtained from (1) the Hubei Statistical Yearbook, Hunan Statistical Yearbook, Jiangxi Statistical Yearbook, and China County Statistical Yearbook; (2) Statistical bulletins on the national economic and social development and government work reports of each city in the corresponding years; (3) Websites of Hubei, Hunan, and Jiangxi cultural and tourism departments.

The dots and polygons data of administrative divisions of China at a scale of 1:1,000,000 were obtained from National Catalogue Service For Geographic Information (http://www.webmap.cn/commres.do?method=result100W), and the administrative division information comes from the query website of the national administrative division information platform of the Ministry of Civil Affairs of the People’s Republic of China (http://202.108.98.30/map).

### Research framework

**Fig 2:**
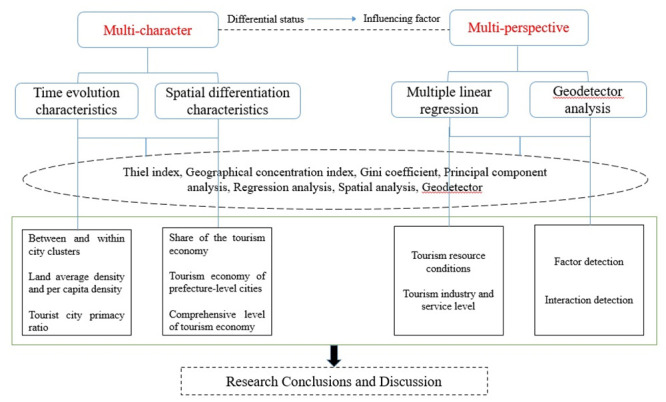
The research framework.

The tourism economy is the touchstone and barometer for measuring tourism development. In order to explore the temporal and spatial patterns as well as influencing factors of tourism economic development differences, a multi-feature, multi-perspective spatiotemporal analysis framework was constructed. This framework includes features of temporal evolution and spatial differentiation, perspectives from multiple linear regression, and perspectives from geodetector (Fig 2). The analysis reveals the temporal evolution characteristics and spatial differentiation characteristics of tourism economic differences among the three major city clusters along the middle reaches of the Yangtze River from 2004 to 2019. Through the use of multiple linear regression, the study dissects the main factors influencing the spatiotemporal differences in tourism economy among these city clusters. The goal is to provide insights for optimizing the strategic layout of tourism in the three major city clusters along the Yangtze River and achieving high-quality coordinated development of regional tourism economy.

### Research methods

#### Single index measurement method

*Theil index*. The Thiel index (T) is a method of regional variation analysis capable of spatial decomposition, which can be divided into two components: intra-group and inter-group variation [28], and can analyze the overall variation in regional variation, the variation in inter- and intra-regional variation, and the impact of the variation in inter- and intra-regional variation on total regional variation. To focus on the characteristics of the economic aspects of tourism, the article uses two indicators of total regional tourism revenue and GDP. The formula is as follows:

$$\begin{array}{l} \text{T}={\text{T}}_{\text{b}}+{\text{T}}_{\text{w}}=\text{Tb}+\sum {\text{T}}_{\text{w}\left(\text{i}\right)}\\ \text{Tb}=\sum \frac{{\text{Y}}_{\text{i}}}{\text{Y}}\times \text{ln}\left(\frac{{\text{Y}}_{\text{i}}}{\text{Y}}/\frac{{\text{G}}_{\text{i}}}{\text{G}}\right)\\ {\text{T}}_{\text{w}\left(\text{i}\right)}=\sum \frac{{\text{Y}}_{\text{ij}}}{{\text{Y}}_{\text{i}}}\times \text{ln}\left(\frac{{\text{Y}}_{\text{ij}}}{{\text{Y}}_{\text{i}}}/\frac{{\text{G}}_{\text{ij}}}{{\text{G}}_{\text{i}}}\right) \end{array}$$ (1)

In the formula: i denotes city clusters (i = 1, 2, 3, corresponding to Wuhan city circle, Chang-Zhu-Tan city group, and Poyang Lake ecological economic zone, respectively); j denotes cities of three major city clusters along the middle reaches of Yangtze River; T is the total regional variation; T_b_ is the inter-regional variation; T_w_ is the intra-regional variation, which is the weighted sum of the intra-regional variation T_w(i)_; Y and G denote the total tourism revenue and GDP values of the three major city clusters along the middle reaches of Yangtze River, Y_i,_ and G_i_ denote the total tourism revenue and GDP values of city clusters i, Y_ij_ and G_ij_ denote the tourism revenue and GDP values of city j of city clusters i respectively. The larger the T, the larger the tourism economic difference; the smaller the T, the smaller the tourism economic difference.

*Geographical concentration index*. The geographical concentration index (G) can be used to measure the concentration of the spatial distribution of the research object [29]. The formula is as follows:

$$\text{G}=100\times \sqrt{\sum_{\text{i}=1}^{\text{n}}{\left({\text{P}}_{\text{j}}/\text{P}\right)}^{2}}$$ (2)

In the formula: G represents the geographical concentration of total tourism income, P_j_ refers to the total tourism income of the jth city of the three city clusters along the middle reaches of the Yangtze River, P refers to the total tourism income of the three city clusters along the middle reaches of the Yangtze River, and n refers to the total number of cities in the three city clusters along the middle reaches of the Yangtze River. The value range of the geographical concentration index G is [0,100]. The larger G is, the more concentrated the city distribution of the total tourism income is; The smaller G is, the more dispersed the city distribution of the total tourism income is.

*Gini coefficient*. The Gini coefficient (G) is mainly used to calculate the balanced (unbalanced) situation of economic development and income distribution and can be used to indicate the concentration degree of spatial distribution in each region. The formula is as follows:

$$\begin{array}{ll} \text{G} & =\frac{2}{\text{n}}\left({\text{k}}_{1}+2{\text{k}}_{2}+\cdots +{\text{nk}}_{\text{n}}\right)-\left(\frac{\text{n}+1}{\text{n}}\right)\\ {\text{G}}_{\text{a}} & =\sum_{\text{a}}\frac{{\text{B}}_{\text{a}}}{\text{B}}{\text{C}}_{\text{a}}\sum_{\text{a}}{\text{D}}_{\text{a}}{\text{C}}_{\text{a}} \end{array}$$ (3)

In the formula: G represents the Gini coefficient; N represents the number of municipal administrative units, n = 23; K_i_ is the proportion of the total tourism economic income of the ith city in the total tourism economic income of the three major city clusters after ascending order; G_a_ is the decomposition share of the Gini coefficient of sub-item a; B is the total economic income of tourism; B_a_ is the economic income of sub-item a; C_a_ is the Gini coefficient of sub-item a; D_a_ is the proportion of sub-item an income to the total tourism income; The contribution rate of sub-item a to tourism economy passed D_a_ C_a_/G×100%.

#### Comprehensive index measurement method

In the study, the commonly used comprehensive index evaluation method is the principal component analysis method, so this study uses it to comprehensively measure the spatial differentiation characteristics of the tourism economy of the three city clusters along the middle reaches of the Yangtze River.

*Index system construction*. The measurement of tourism economic development differences always requires certain indicators, and these indicators should be able to reflect the overall situation of tourism development in different regions. At present, the most used indicators to evaluate the development of the tourism economy are international tourism income, domestic tourism income, total tourism income, number of inbound tourists, number of domestic tourists, and the total number of tourists. Tourism is a highly related industry. In addition to the six indicators mentioned above, the indicators related to the development of the tourism economy include the number of one-day tourists, the number of per capita stay days, the number of travel agencies, the number of star hotels, the number of class-A tourist attractions, tourism fixed assets investment, the number of tourism employment, the quality of tourist attractions, the number of tourism students in school, etc.

The selection of indicators determines the content of the evaluation, and the reasonableness of indicator selection directly impacts the objectivity, comprehensiveness, and scientific validity of the measurement results. To best reflect the current situation of tourism economic development of the three major city clusters along the middle reaches of the Yangtze River, under the premise of ensuring the availability of data and the comparability between regions, and based on the principles of scientificity, comprehensiveness, operability, hierarchy, and quantification, and summarizing the evaluation indicators and methods of other scholars [30, 31], the comprehensive indicator system (Table 1) is drawn, which is divided into two levels: 6 primary indicators, 14 secondary indicators.

**Table 1:** Comprehensive evaluation index system of tourism economic development difference.

Primary indicators	Secondary indicators	Unit
Tourism economic income	Domestic tourism income	100million CNY
	International tourism income	million USD
Tourist reception number	Number of domestic tourists	10000 person-time
	Number of Inbound tourists	10000 person-time
Current situation of tourism resource	Number of class-A tourist attractions	pcs
	Proportion of tourist attractions above 4A	%
Current situation of tourism industry	travel agencies	pcs
	Star hotels	pcs
Tourism economic status	Proportion of tourism income in local GDP	%
	Proportion of tourism revenue in the tertiary industry	%
Tourism development potential	GDP growth rate	%
	GDP per capita	CNY
	Per capita disposable income of urban residents	CNY
	Per capita disposable income of rural residents	CNY

*Principal component analysis*. It transforms several sample variables into several comprehensive variables (i.e., principal components) through a linear transformation through dimensionality reduction. The principal components are all linear combinations of the original variables and are not related to each other. Therefore, these comprehensive variables can reflect most of the information of the original variables, and the information reflected does not overlap with each other [32]. According to this principle and formula, the combined score value of the principal components F.

#### Geodetector method

The geodetector is a flexible and convenient new statistical method designed to detect spatial variations and their influencing factors. It enables the measurement of spatial variations in given data, identifies the variables with the maximum spatial differences, and explores the variables that explain the dependent variable. Additionally, it quantifies the interactive effects of pairwise factors on the dependent variable. This method finds extensive applications in relevant studies within the field of socioeconomics. The formula is as follows [33]:

$$\text{q}=\left({\text{N}\sigma }^{2}-\overset{\text{L}}{\underset{\text{h}=1}{\sum }}{\text{N}}_{\text{h}}\sigma_{\text{h}}^{2}\right)/{\text{N}\sigma }^{2}$$ (4)

In the formula: where h = 1, 2,…, L represents the number of categories for the h-th class of influencing factors; N and σ^2^ are the sample size and variance of the whole region, respectively; N_h_ and $\sigma_{h}^{2}$ are the sample size and variance for the h-th class of influencing factors. q is the explanatory strength of the independent variable for the spatial variance of the dependent variable, and the domain of the value of q is [0, 1], and the greater the value of q, the greater the ability of the independent variable to explain the spatial divergence of the dependent variable, and vice versa.

## Results and analysis

### The temporal evolution characteristics

#### Temporal evolution characteristics between and within city clusters

*Variance change analysis*. To better reveal the dynamics of tourism economic development disparities among the three major city clusters along the middle reaches of the Yangtze River, as well as the intra-regional and inter-group differences in both visitors and income concentration, a thorough analysis is conducted using the Theil index and geographical concentration index.

From the perspective of theil coefficient (Table 2), the overall difference between the tourism economy of the three major city clusters along the middle reaches of the Yangtze River and the fluctuation of differences within each city cluster decreased, and the fluctuation of differences between groups increased. From 2004 to 2019, the difference between the Chang-Zhu-Tan city group decreased the most, and the difference is the smallest at present. The difference of Poyang Lake ecological economic zone is the most stable, and the difference is relatively small. The difference between Wuhan city circle has decreased significantly, and the difference is currently the largest.

**Table 2:** Theil coefficient and geographical concentration index of tourism economic differences among the three major city clusters along the middle reaches of the Yangtze River.

Year	Theil coefficient					Geographical concentration index		
	Intra-group differences			Inter-group differences	Overall differences	Domestic tourism	International tourism	Total tourism income
	Wuhan city circle	Chang-Zhu-Tan city group	Poyang Lake ecological economic zone					
**2004**	0.1802	0.1811	0.1095	0.0034	0.4707	38.70	53.26	39.23
**2005**	0.1562	0.1253	0.1066	0.0087	0.3881	38.08	54.44	38.72
**2006**	0.1469	0.0399	0.1284	0.0030	0.3152	35.51	52.40	36.11
**2007**	0.1178	0.0600	0.1259	0.0025	0.3038	35.21	50.46	35.76
**2008**	0.1154	0.0298	0.1093	0.0031	0.2546	34.77	49.42	35.22
**2009**	0.0990	0.0257	0.1086	0.0036	0.2333	35.46	48.07	35.84
**2010**	0.1123	0.0227	0.1289	0.0089	0.2639	36.73	48.93	37.08
**2011**	0.1372	0.0115	0.0834	0.0135	0.2321	38.04	49.22	38.31
**2012**	0.1457	0.0084	0.0824	0.0170	0.2365	38.55	52.87	38.90
**2013**	0.1466	0.0062	0.0758	0.0188	0.2286	37.83	54.10	38.15
**2014**	0.1398	0.0079	0.0758	0.0321	0.2234	36.60	54.86	36.89
**2015**	0.1271	0.0102	0.0678	0.0471	0.2051	34.66	61.97	35.05
**2016**	0.1241	0.0197	0.0617	0.0591	0.2055	32.95	64.34	33.35
**2017**	0.1170	0.0257	0.0623	0.0368	0.2050	31.59	60.29	31.95
**2018**	0.1011	0.0223	0.0836	0.0491	0.2069	30.79	59.48	31.10
**2019**	0.1076	0.0189	0.0807	0.0522	0.2072	30.42	60.17	30.77

From the perspective of the geographical concentration index (Table 2), the tourism concentration of the three major city clusters along the middle reaches of the Yangtze River is relatively high, and the overall trend is fluctuating and declining. The geographical concentration of domestic tourism income and total tourism income is almost the same. The geographical concentration of international tourism income is more obvious, with a large fluctuation, and shows a trend of gradual increase. The highest geographical concentration index is 64.34, and the lowest is 48.07. This shows that the tourism economy of the three major city clusters along the middle reaches of the Yangtze River, especially the international tourism economy, is mainly concentrated in several large cities and cities with strong tourism characteristics, that is, Wuhan, Changsha, Yueyang, Jiujiang, Shangrao, Nanchang and Jingdezhen.

*(2) Differential contribution analysis*. The contribution of regional tourism economic differentiation is measured by the Gini coefficient of tourism revenue of the three major city clusters along the middle reaches of the Yangtze River, and the overall differentiation categories are analyzed according to the contribution rates of domestic tourism revenue and international tourism revenue.

From the Table 3, we know that the Gini coefficients of the total tourism economic income of the three major city clusters along the middle reaches of the Yangtze River show a fluctuating decline over time, indicating that the differences in tourism economies of the three major city clusters are fluctuating and narrowing. The Gini coefficient of domestic tourism economic income of the three major city clusters fluctuation decreases significantly, and the Gini coefficient of international tourism income remains stable. Combined with the Gini coefficient contribution rate, the change in domestic tourism income difference determines the change in total tourism income difference, and the changing trend of domestic tourism income coincides with the changing trend of total tourism income.

**Table 3:** Gini coefficient and the contribution rate of tourism economy of three major city clusters along the middle reaches of the Yangtze River.

Year	Gini coefficient			Contribution rate (%)	
	Total tourism economic income	Domestic tourism economic income	International tourism economic income	Domestic tourism economic income	International tourism economic income
**2004**	0.5954	0.5861	0.8212	95.46	4.54
**2005**	0.5953	0.5861	0.8169	95.45	4.55
**2006**	0.5776	0.5675	0.7963	95.14	4.86
**2007**	0.5656	0.5561	0.7803	95.24	4.76
**2008**	0.5512	0.5429	0.7555	95.42	4.58
**2009**	0.5456	0.5386	0.7453	96.09	3.91
**2010**	0.5536	0.5472	0.7461	96.30	3.70
**2011**	0.5558	0.5501	0.7427	96.70	3.30
**2012**	0.5581	0.5535	0.7482	97.33	2.67
**2013**	0.5564	0.5527	0.7515	97.84	2.16
**2014**	0.5497	0.5462	0.7607	98.22	1.78
**2015**	0.5381	0.5342	0.8002	98.14	1.86
**2016**	0.5330	0.5287	0.8136	98.31	1.69
**2017**	0.5233	0.5191	0.8198	98.81	1.19
**2018**	0.5081	0.5044	0.8124	98.35	1.65
**2019**	0.4990	0.4941	0.8149	98.39	1.61

### Temporal evolution characteristics of land average density and per capita density

To eliminate the influence of population and land area on the comparison results, and to show the differences in tourism economy between regions more objectively and comprehensively, we compare the average land density and per capita density of the tourism economy in various regions. The average land density refers to the tourism output value created per 10,000 square kilometers; Per capita density reflects the tourism wealth per 10,000 people.

**Fig 3:**
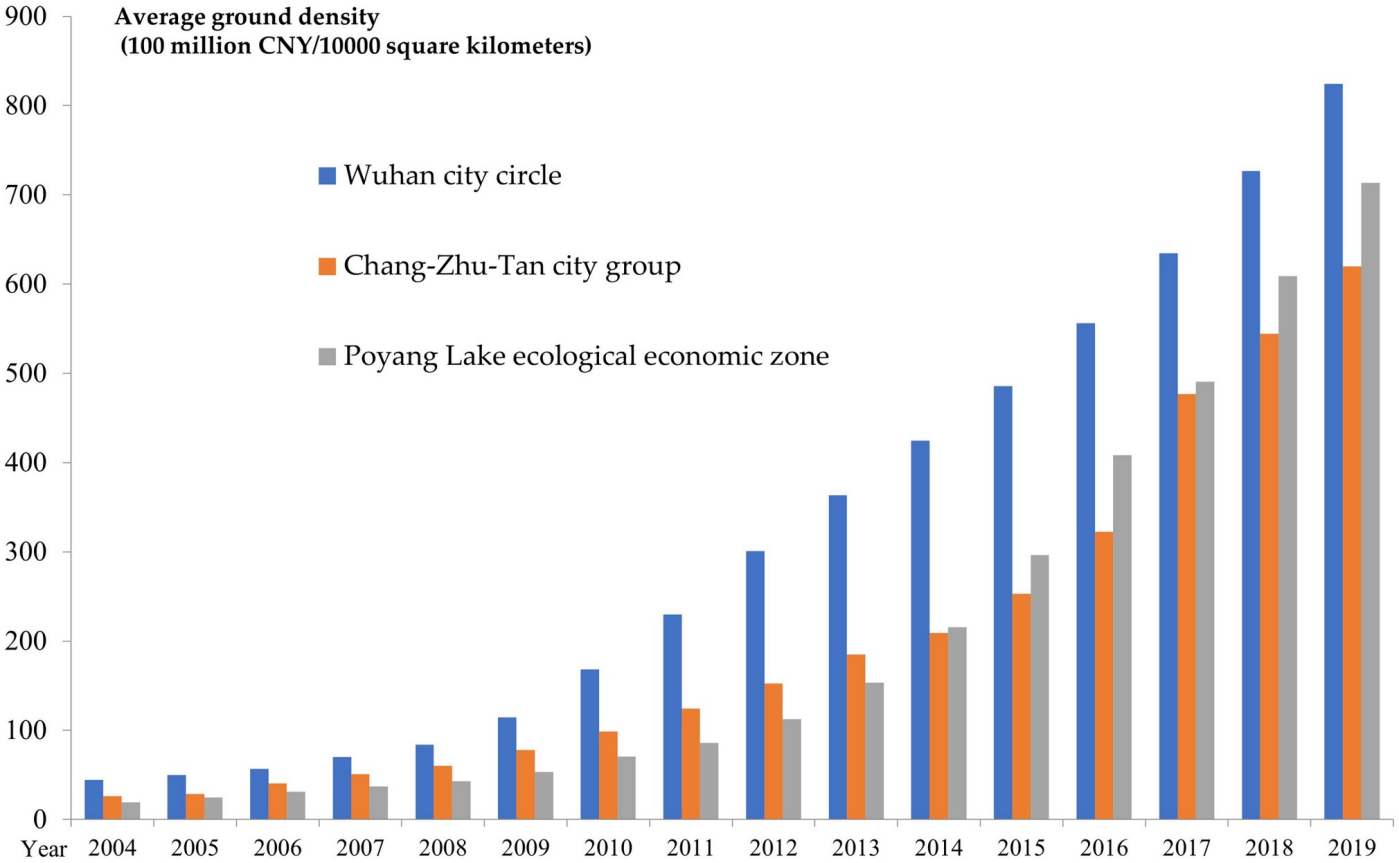
The land average density of the total tourism income. The comparison here is the land average density of total tourism revenue of the three major city clusters along the middle reaches of the Yangtze River, with blue color representing Wuhan city circle, red color representing Chang-Zhu-Tan city group, and gray color representing Poyang Lake ecological economic zone.

It can be seen from Fig 3 that the tourism output value created by Wuhan city circle per 10,000 square kilometers has always ranked first among the three major city clusters, and the tourism economy of Poyang Lake ecological economic zone has developed rapidly, and the average density of land in 2014 exceeded that of the Chang-Zhu-Tan city group. With the evolution of time, the difference in tourism development of the three major city clusters first widened and then narrowed.

**Fig 4:**
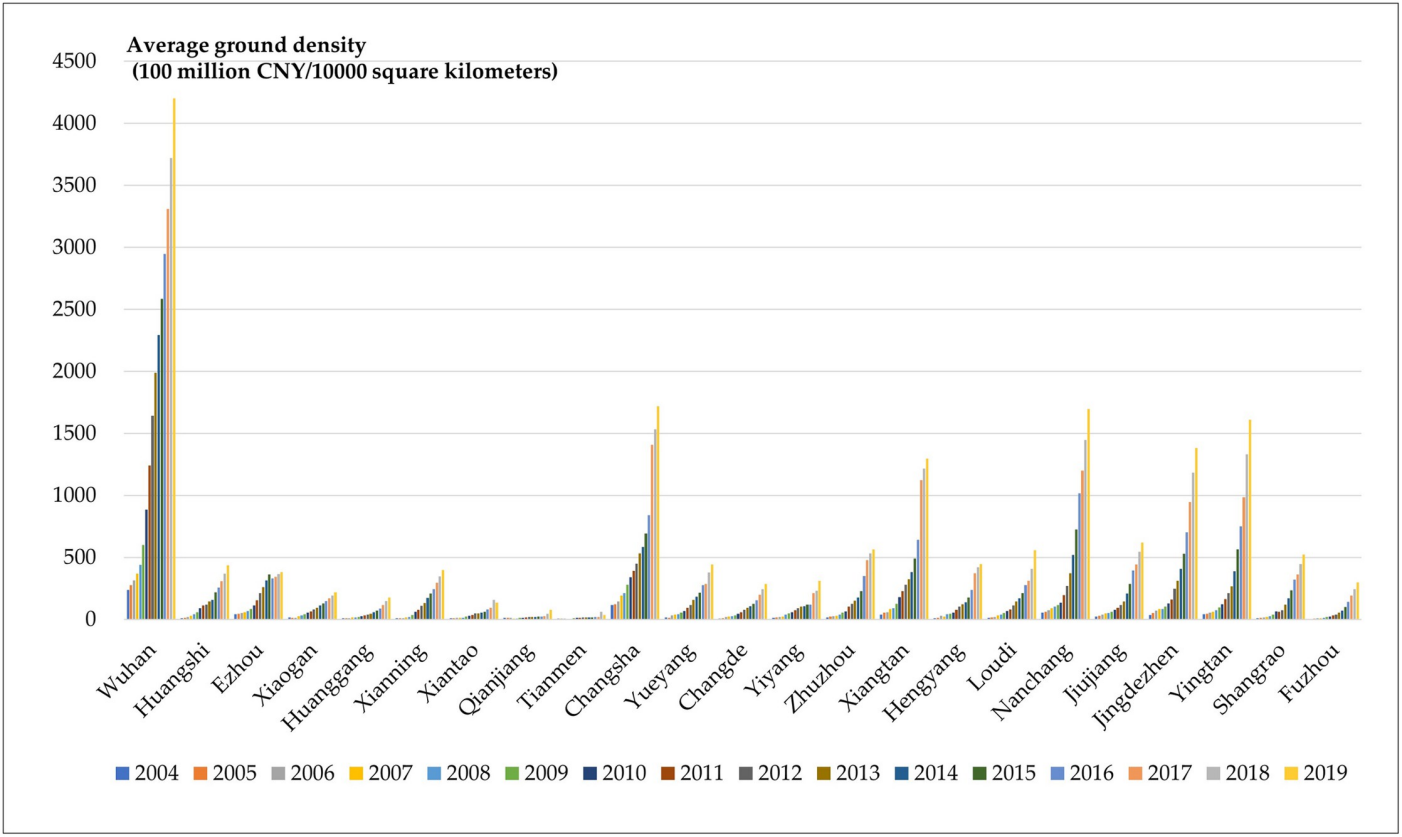
The land average density of the total tourism income of each city.

It can be seen from Fig 4 that Wuhan has consistently ranked first in terms of the land average density, with obvious advantages; Changsha, Nanchang, Yingtan, Jingdezhen, and Xiangtan all exceed 100 billion/10,000 square kilometers; Jiujiang, Huangshi, Xiaogan, Xianning, Yueyang, Changde, Yiyang, Zhuzhou, Hengyang, Loudi and Shangrao are at the third level; The remaining cities create the smallest tourism output value per 10,000 square kilometers, and the tourism industry is weak.

**Fig 5:**
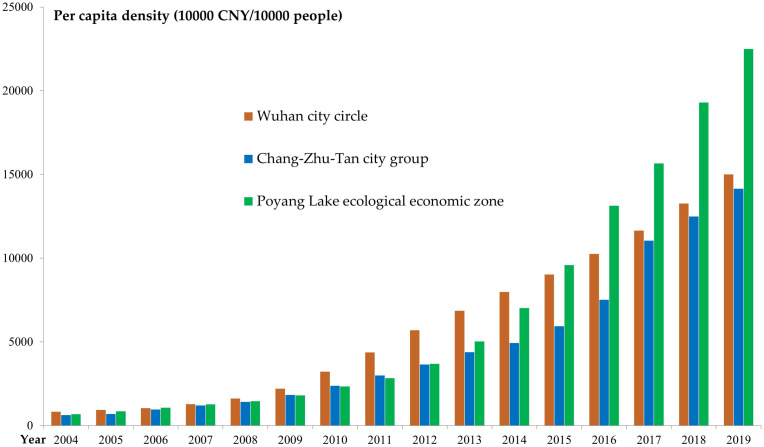
The per capita density of the total tourism income. The comparison here is the per capita density of total tourism revenue of the three major city clusters along the middle reaches of the Yangtze River, with red color representing Wuhan city circle, blue color representing Chang-Zhu-Tan city group, and green color representing Poyang Lake ecological economic zone.

The per capita density of city clusters (Fig 5) showed a trend of "competing development", with Wuhan city circle maintaining first place before 2015, and then the Poyang Lake ecological economic zone surpassed and widened the gap. The Chang-Zhu-Tan city group has the least tourism wealth per 10,000 people, but the development speed is in the middle, and the Wuhan city circle has the slowest development speed.

**Fig 6:**
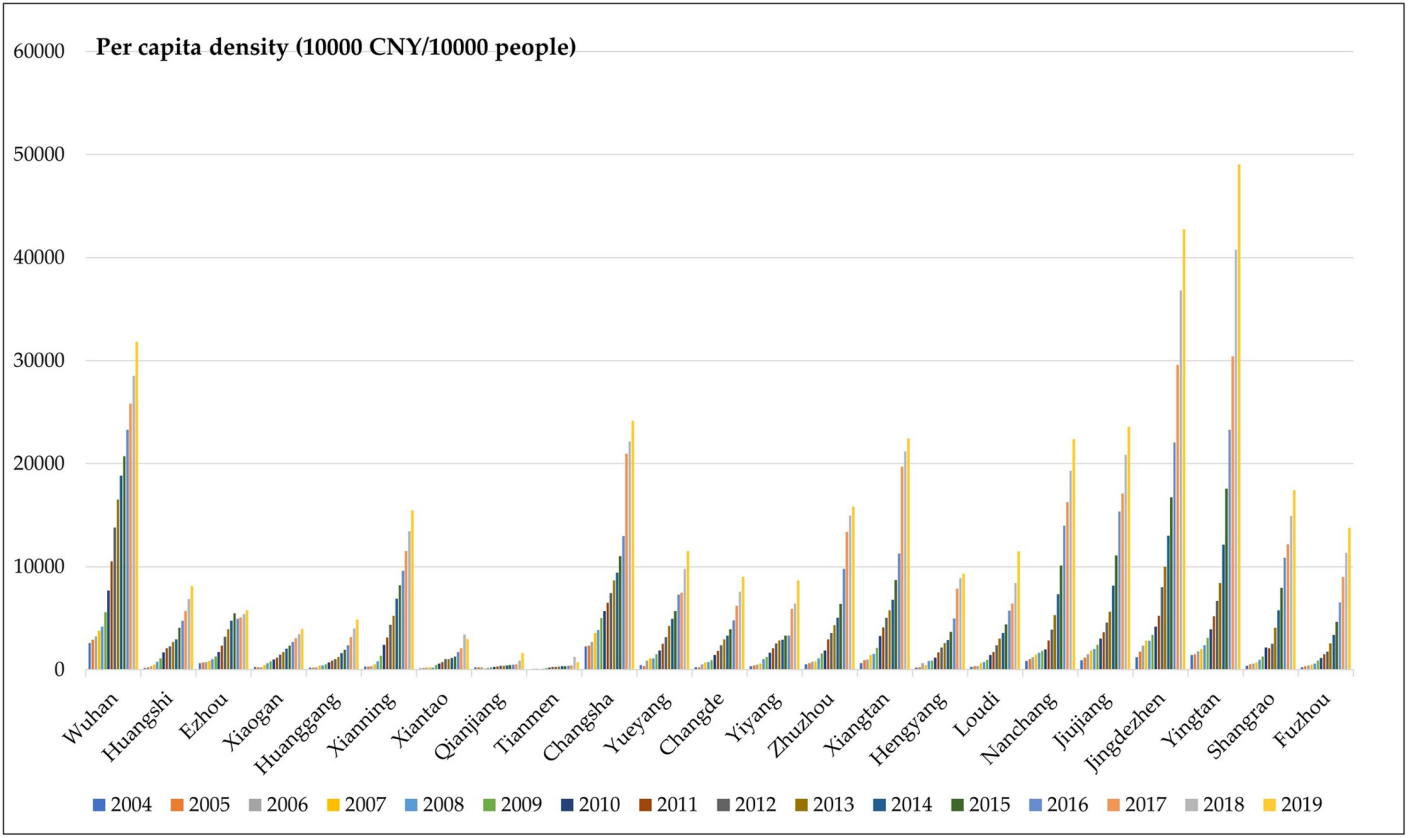
The land average density of the total tourism income of each city.

The per capita density of various cities is consistent with that of urban agglomerations (Fig 6), and the per capita density of Yingtan and Jingdezhen exceeds 40,000 yuan, ranking in the first group; Wuhan, Changsha, Jiujiang, Nanchang, and Xiangtan are in the second group, with a per capita density of more than 20,000 yuan; Xianning, Yueyang, Zhuzhou, Loudi, Shangrao, and Fuzhou are in the third group; The per capita density of the remaining cities is less than 10,000 yuan, and the per capita tourism wealth is the smallest.

The tourism economy of the three major city clusters along the middle reaches of the Yangtze River continues to develop, but there are great differences in the tourism economy of each region. In terms of city clusters: the three major city clusters have their characteristics, and in terms of total volume, the Chang-Zhu-Tan city group began to rank first in 2017; In terms of average land density, Wuhan city circle has always been the first, but since 2015, the advantage has been shrinking; In terms of per capita density, the Poyang Lake ecological economic zone jumped to the top in 2015 and has continued to expand its advantages since then. In terms of cities: in terms of total volume, Wuhan, Changsha, Nanchang, Jiujiang, and Shangrao are the largest, and Xiaogan, Ezhou, Xiantao, Qianjiang, and Tianmen in Wuhan city circle are the smallest; In terms of average land density, Wuhan has always maintained the first place, and Changsha, Nanchang, Yingtan, Jingdezhen and Xiangtan are in the second echelon; In terms of per capita density, Yingtan and Jingdezhen began to rank in the top 2 in 2017. Overall, the difference in tourism economy has been taken as the critical point around 2015, showing a trend of "first expanding and then shrinking", and this difference is not random, but hierarchical. The contribution rate of the domestic tourism economy to the overall tourism economy exceeds 90% and narrowing the difference in the tourism economy mainly lies in narrowing the gap between domestic tourism economies in various cities in the city clusters.

### Temporal evolution characteristics of tourist city primacy ratio

**Fig 7:**
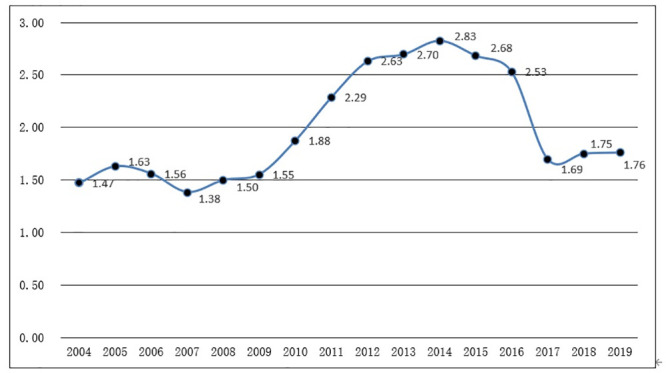
The primacy ratio of tourism economy of the three major city clusters along the middle reaches of the Yangtze River.

Tourist city primacy ratio is transplanted from the concept of city primacy ratio in urban geography. It refers to the ratio of the scale of the tourism economy between the first and second places. Generally, it is used to indicate the tourism scale structure and the level of tourist concentration in a region, using the indicator of total tourism revenue in this case. From 2004 to 2019, the top two cities in terms of tourism revenue in the three major city clusters along the middle reaches of the Yangtze River were consistently Wuhan and Changsha. The primacy ratio showed a "inverted U-shaped" trend over time (Fig 7). From 2004 to 2007, the primacy ratio was gradually decreasing, reaching its lowest value of 1.383 in 2007. This indicates a narrowing of economic differences in the developed areas of the tourism economy in the three major city clusters along the middle reaches of the Yangtze River. From 2008 to 2012, the primacy ratio continued to expand, surpassing 2 in 2011 and reaching a high value of 2.83 in 2014. According to general rules, a primacy ratio greater than 2 indicates a trend towards structural imbalance and excessive concentration, suggesting that the leading tourism city in the three major city clusters along the middle reaches of the Yangtze River is prominent, with a risk of gradual structural imbalance. Afterward, the primacy ratio rapidly declined and stabilized around 1.7 in recent years, indicating a gradual normalization of the structure.

### The spatial difference characteristics

#### Spatial difference characteristics in the share of the tourism economy

**Fig 8:**
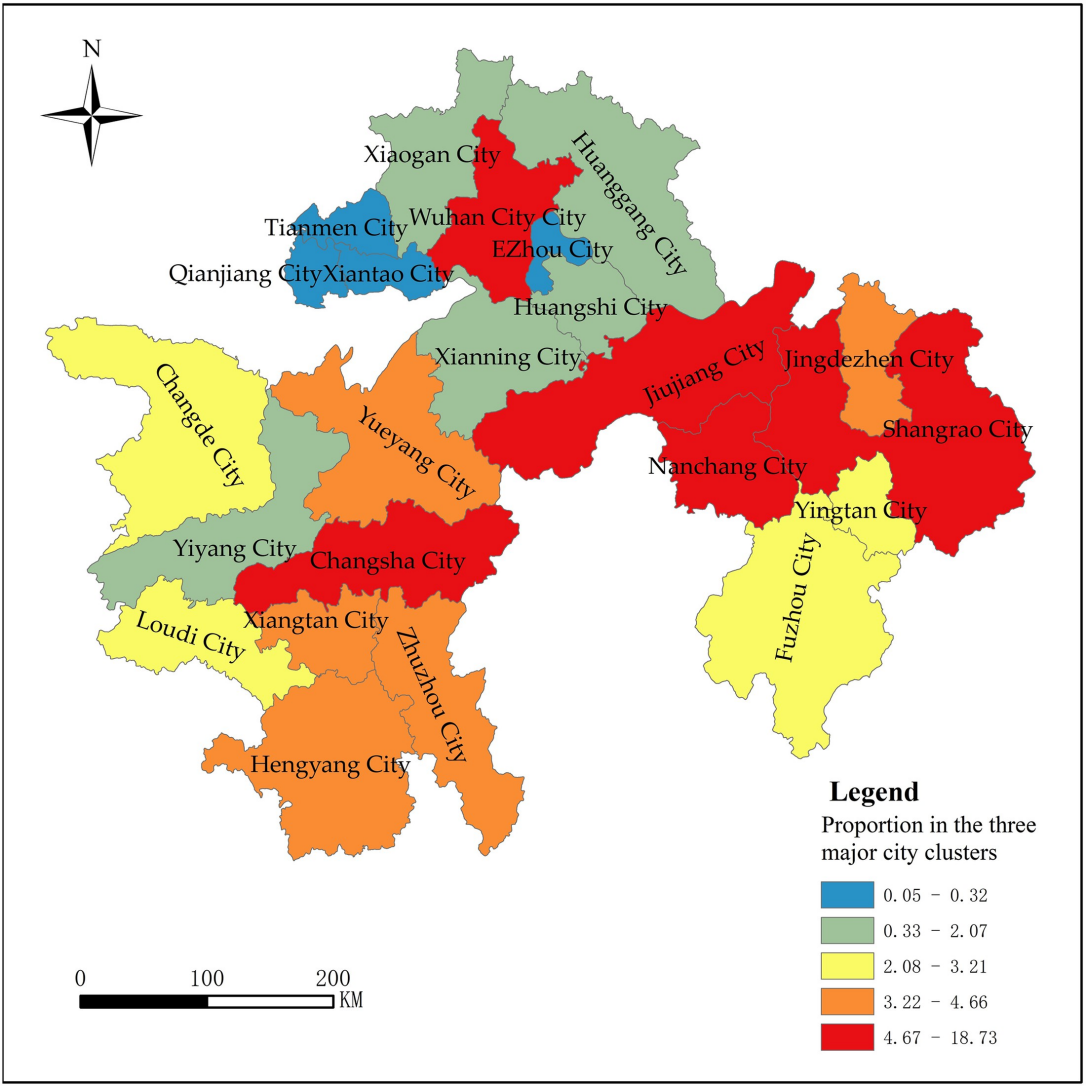
The total tourism revenue of each city in the proportion of the three major city clusters along the middle reaches of the Yangtze River (2019).

**Fig 9:**
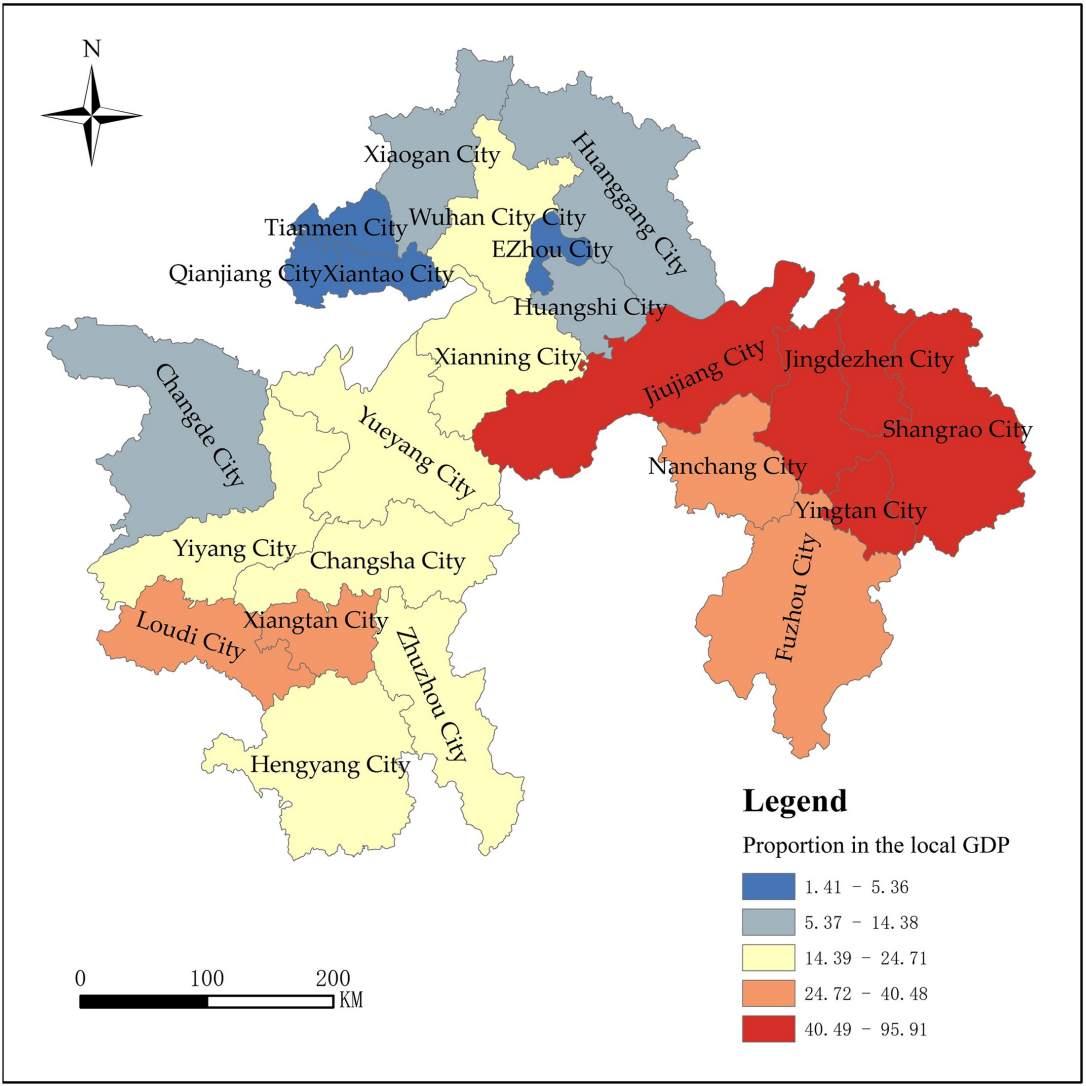
The proportion of total tourism revenue to local GDP in each city (2019).

Figs 8 and 9 shows that in terms of the total tourism economy, Wuhan, Shangrao, Jiujiang, Changsha, Nanchang, and Jingdezhen each account for over 4% of the total tourism income of the three major city clusters in the middle reaches of the Yangtze River; The total tourism economic volume of Ezhou, Xiantao, Qianjiang, and Tianmen cities is low, and their share of tourism revenue in the three major city clusters in the middle reaches of the Yangtze River does not exceed 1%. From the perspective of tourism economic status, Jingdezhen, Shangrao, Jiujiang, Yingtan, Fuzhou, Nanchang, Xiangtan, and Loudi account for a relatively high proportion, with tourism income accounting for over 27% of local GDP, becoming a pillar industry for local economic development, and playing a pivotal role in regional development; The proportion of Xiantao, Qianjiang, and Tianmen cities is relatively low, with tourism revenue accounting for less than 5% of local GDP, and Qianjiang, Tianmen, and even less than 2%, and the contribution of tourism to the regional economy is low.

#### Spatial difference characteristics in the tourism economy of prefecture-level cities

**Fig 10:**
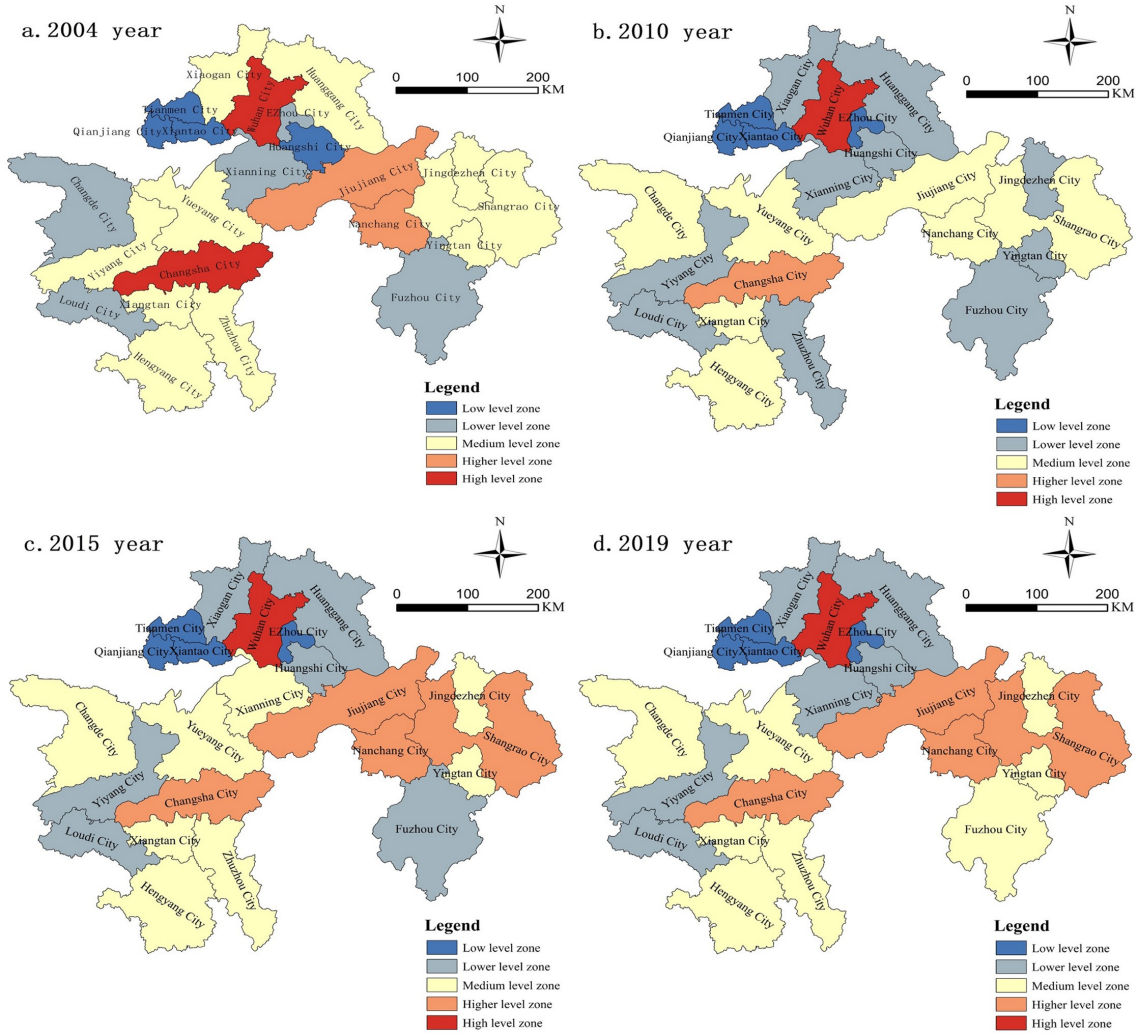
Prefecture-level cities spatial distribution of tourism economic development differences.

In order to deeply analyze the spatial characteristics of the differences in tourism economic development among the three major city clusters along the middle reaches of the Yangtze River, ArcGIS spatial analysis tools were employed. Using the natural breaks method and based on the total tourism revenue of each city, the cities were categorized into five levels of scale. The resulting map illustrates the spatial distribution of tourism economic development differences in the three major city clusters along the middle reaches of the Yangtze River (Fig 10). In 2004, the percentages of cities in the low, relatively low, moderate, relatively high, and high-scale levels were 17.39%, 21.47%, 43.48%, 8.7%, and 8.7%, respectively. By 2010, the percentages changed to 17.39%, 43.48%, 30.43%, 4.35%, and 4.35%. In 2015, they were 17.39%, 26.09%, 34.78%, 17.39%, and 4.35%, and in 2019, the percentages were 17.39%, 26.09%, 34.78%, 17.39%, and 4.35%. Overall, the proportion of high and relatively high-scale tourism economies decreased from 17.4% in 2004 to 8.7% in 2010, and then expanded to 21.74% in 2019. This suggests that the tourism economic differences among the cities in the three major city clusters along the middle reaches of the Yangtze River exhibited a pattern of "expansion followed by contraction".

Tourism economy is positively correlated with city size and the number of class-A tourist attractions. Wuhan, with abundant class-A tourist attractions and being a provincial capital city, consistently remained in the high-level zone. Cities with smaller populations and fewer class-A tourist attractions, such as Tianmen, Qianjiang, and Xiantao, consistently remained in the low-level zone. By 2019, cities in the high and relatively high-level zones included Wuhan (provincial capital), Changsha (provincial capital), Nanchang (provincial capital), Jiujiang, and Shangrao, where there were more class-A tourist attractions. The moderate-level zone included cities such as Yueyang, Changde, Xiangtan, Zhuzhou, Hengyang, Fuzhou, Yingtan, and Jingdezhen. The remaining cities were mainly in the relatively low and low-level zones, mostly surrounding high and relatively high-level cities. Specifically, more than 78% of cities in the three major city clusters along the middle reaches of the Yangtze River have tourism economic development at the medium to low levels. Although some cities evolved from low to moderate levels, only Shangrao entered the relatively high-level zone by 2019.

#### Spatial difference characteristics in the comprehensive level of the tourism economy

To strengthen the comprehensive analysis of the tourism differences between the three major city clusters along the middle reaches of the Yangtze River, according to Table 1, we process and analyze the relevant data of the three major city clusters along the middle reaches of the Yangtze River in 2019 by SPSS20.0 and EXCEL2010. Next, we extract the principal components and rotate the principal component factors to obtain Table 4.

**Table 4:** Rotate the principal component matrix.

Indicator	Principal Component			
	F_1_	F_2_	F_3_	F_4_
International tourism income	.844	.413	.233	.038
Number of Inbound tourists	.826	.415	.272	.163
Proportion of tourist attractions above 4A	.690	.163	.047	.413
Domestic tourism income	.635	.409	.548	.297
Number of domestic tourists	.630	.341	.569	.324
Per capita disposable income of rural residents	.128	.892	.067	-.205
GDP per capita	.245	.892	.056	-.177
Per capita disposable income of urban residents	.304	.840	.307	.111
GDP growth rate	-.636	.576	-.261	.525
Number of class-A tourist attractions	.090	-.033	.908	.037
Star hotels	.353	.203	.809	.262
travel agencies	.228	.517	.711	.174
Proportion of tourism income in local GDP	.211	-.158	.224	.901
Proportion of tourism revenue in the tertiary industry	.155	-.181	.209	.896

The first principal component (F_1_) has a large load on the international tourism income, the number of inbound tourists, the proportion of tourist attractions above 4A, the domestic tourism income, and the number of domestic tourists, which are 0.844, 0.826, 0.690, 0.635, and 0.630, respectively. Among them, indicators such as international tourism income, number of inbound tourists, domestic tourism income, and number of domestic tourists reflect the development of the tourism economy. The proportion of tourist attractions above 4A reflects the quality of tourism resources, which has important relevance to the development of the tourism economy. The second principal component (F_2_) has a strong load on the per capita disposable income of rural residents, GDP per capita, per capita disposable income of urban residents, and GDP growth rate, which are 0.892, 0.892, 0.840, and 0.576, respectively. These four indicators reflect the development potential and supporting capacity of the tourism economy.

The third principal component (F_3_) has a high load on the number of class-A tourist attractions, star hotels, and travel agencies, which are 0.908, 0.809, and 0.711, respectively. The number of class-A tourist attractions represents the richness of tourism resources, and the number of star hotels and travel agencies reflects the development of the tourism industry.

The fourth principal component (F_4_) has a large load on the proportion of tourism income in local GDP and the proportion of tourism revenue in the tertiary industry, which are 0.901 and 0.896, respectively. These two indicators reflect the position of the tourism economy in the national economy.

According to the comprehensive index measurement method, the comprehensive score value and ranking of the tourism economy of 23 cities in the three major city clusters along the middle reaches of the Yangtze River are calculated, and the calculation results are shown in Table 5.

**Table 5:** A comprehensive evaluation of the tourism economy of the three major city clusters along the middle reaches of the Yangtze River in 2019.

City	The first principal component(F_1_)	The second principal component (F_2_)	The third principal component (F_3_)	The fourth principal component (F_4_)	Comprehensive score(F)	Ranking
Wuhan	3.65	1.39	0.61	-1.04	1.36	1
Changsha	-0.42	2.92	1.35	-0.36	0.94	2
Shangrao	1.82	-0.89	-0.05	2.37	0.73	3
Jiujiang	-0.23	-0.20	1.81	1.53	0.62	4
Nanchang	-0.64	0.82	1.40	0.26	0.43	5
Jingdezhen	0.51	-0.15	-0.83	2.22	0.34	6
Zhuzhou	-0.22	0.83	-0.65	0.18	0.04	7
Yingtan	-0.65	0.13	-0.49	1.20	-0.02	8
Xiangtan	-0.21	0.51	-0.74	0.26	-0.05	9
Fuzhou	-1.21	-0.77	1.95	0.10	-0.06	10
Yueyang	-0.22	-0.27	0.22	-0.29	-0.14	11
Hengyang	-0.58	-0.11	0.23	-0.26	-0.19	12
Xianning	-0.19	-0.44	-0.19	0.08	-0.21	13
Huangshi	-0.88	0.30	0.09	-0.38	-0.22	14
Changde	-0.24	-0.34	-0.12	-0.32	-0.26	15
Ezhou	-0.50	0.66	-1.03	-0.57	-0.33	16
Xiaogan	-0.35	-0.29	-0.43	-0.43	-0.37	17
Loudi	-0.04	-1.03	-0.49	0.12	-0.39	18
Huanggang	0.50	-1.97	1.33	-1.64	-0.40	19
Qianjiang	-0.07	0.34	-1.55	-0.53	-0.41	20
Xiantao	-0.33	0.29	-1.18	-0.57	-0.41	21
Yiyang	0.67	-1.51	0.04	-1.30	-0.48	22
Tianmen	-0.18	-0.22	-1.28	-0.62	-0.55	23

**Fig 11:**
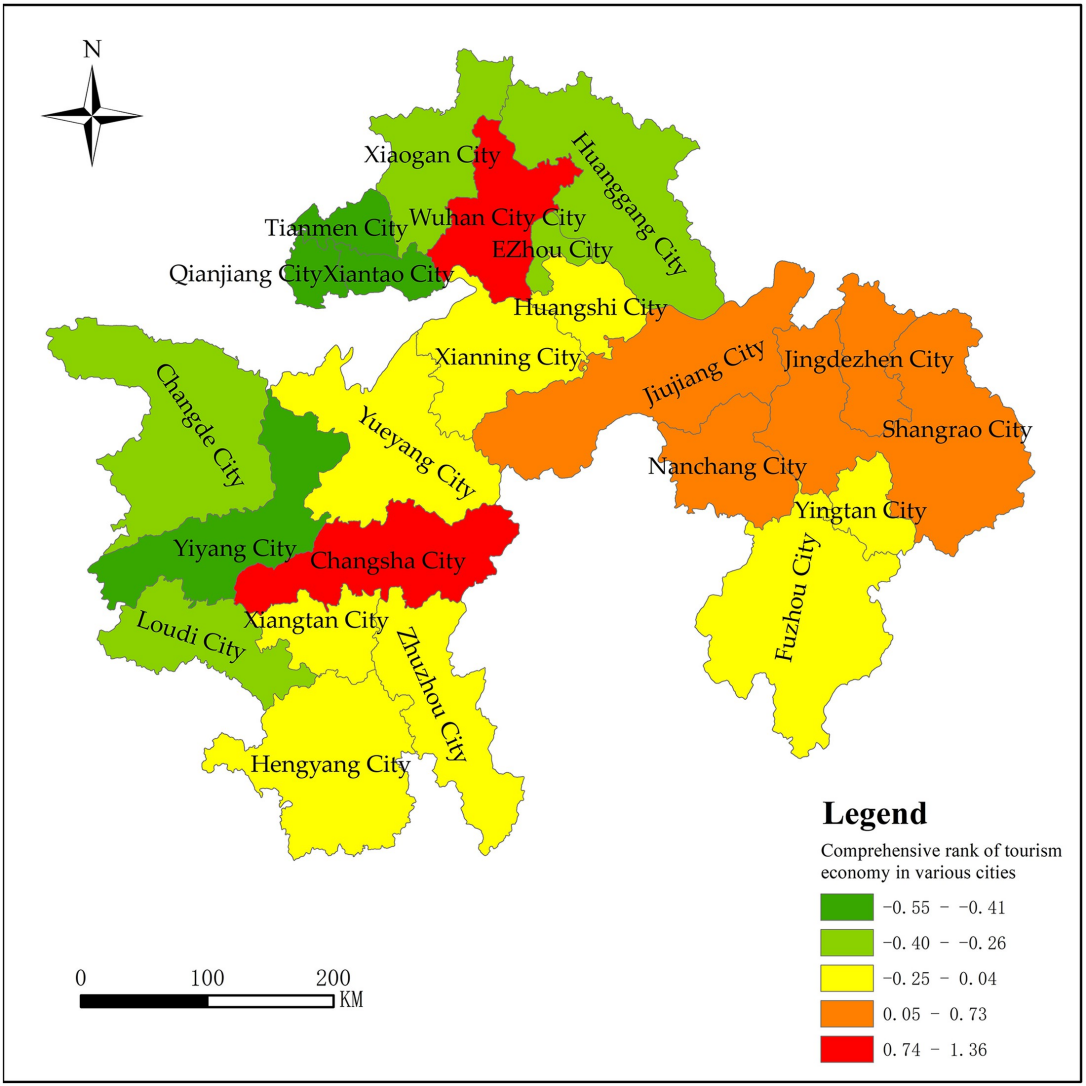
Comprehensive differences of tourism economy in the three major city clusters along the middle reaches of the Yangtze River (2019).

From Table 5, there are 7 cities with F greater than zero and 16 cities with F less than zero in the three major city clusters along the middle reaches of the Yangtze River. Combined with the evaluation results of the spatial differences in the tourism economy and the cluster analysis results, the natural breakpoint method is used in ArcGIS 10.7 to divide the differences in tourism economic development of the three major city clusters along the middle reaches of the Yangtze River into five levels, and the results are shown in Fig 11.

The first level: Wuhan and Changsha. The comprehensive evaluation scores of Wuhan and Changsha were 1.36 and 0.94 respectively, and much higher than other cities in the three major city clusters along the middle reaches of the Yangtze River. The principal components F_1_, F_2,_ and F_3_ in Wuhan are 3.65, 1.39, and 0.61, respectively, indicating that Wuhan has significant advantages in terms of tourism economic development, tourism industry development, tourism resource richness, and tourism economic development potential. Changsha has scored positive on F_2_ and F_3_, respectively 2.92 and 1.35, and ranked first on the principal component F_2_, indicating that the city’s tourism development has received widespread support, benefiting from local economic and social development, and has great potential for tourism economic development.

The second level: is Shangrao, Jiujiang, Nanchang, and Jingdezhen. The comprehensive tourism economic scores of these four regions are 0.73, 0.62, 0.43, and 0.34, respectively. Shangrao’s score on the principal component F_4_ ranks first among the three major city clusters along the middle reaches of the Yangtze River, indicating that the city’s tourism economy occupies a high position in the national economy and tourism is a pillar industry for local economic development. Jingdezhen also scored high on the principal component F_4_, reaching 2.22, reflecting the relatively important position of tourism in the economic and social development of Jingdezhen. Jiujiang has scored higher on the principal component F_3_, reaching 1.81, reflecting its rich tourism resources and high development level of the tourism industry. Nanchang has three main components with positive scores, and the development of the tourism economy is relatively balanced. However, efforts need to be made to improve the contribution of the tourism economy and the quality of tourism resources.

The third level: is Zhuzhou, Yingtan, Xiangtan, Fuzhou, Yueyang, Hengyang, Xianning, and Huangshi. The number of cities in this level is the largest, and the comprehensive score is concentrated between -0.25 and 0.04. The overall development level of the tourism economy is average, but there are certain differences in specific scores. There are two positive main components in Zhuzhou, Yingtan, Xiangtan, Fuzhou, and Huangshi, respectively. Among them, Zhuzhou and Xiangtan have strong support forces for tourism development and great potential for tourism development; Yingtan has a high tourism economic status. Yueyang, Hengyang, and Xianning have only one positive principal component, and Yueyang and Hengyang have relatively good development in terms of the number of tourism resources and the tourism industry. Overall, cities at this level have negative scores on the principal component F_1_, indicating that the overall tourism economy of cities at this level is low and the quality of tourism resources is weak.

The fourth level: Changde, Ezhou, Xiaogan, Loudi, Huanggang. The comprehensive scores of the five cities are all negative, concentrated between -0.26 and -0.40, and the overall development level of the tourism economy is relatively low. From the specific scores of the four principal components, Huanggang has 2 positive scores, Ezhou and Loudi have 1 positive score, and Changde and Xiaogan have negative scores. Huanggang scored 1.33 on F_3_, indicating that it has certain advantages in terms of the number of tourism resources. Ezhou scored 0.66 on F_2_, indicating that it has certain advantages in terms of tourism economic development potential. Overall, these five cities have relatively low levels of tourism economic development performance, industrial status, supporting forces, and tourism resource quality.

The fifth level: Qianjiang, Xiantao, Yiyang, Tianmen. Their comprehensive evaluation scores are -0.41, -0.41, -0.48, and -0.55, respectively, which are the three major city clusters along the middle reaches of the Yangtze River with the most backward tourism economic development. The F_4_ scores of the four cities are all negative, indicating that the tourism economy has a low status and insufficient contribution to local economic development. In most cities, only one item score is positive, and the four principal component scores of Tianmen are all negative.

## Influencing factors of the differences in tourism economic development

The factors affecting regional tourism development are wide and complex [34]. These different factors jointly restrict the development of the regional tourism economy, and academics believe that the factors affecting tourism economic differences mainly include tourism resource conditions, economic development level, tourism industry and service level, industrial structure and location conditions [35]. Tourism resources constitute the core elements driving regional tourism economic development and form the mainstay of tourism destination development and construction. The level of regional economic development provides the objective material foundation for the development of tourism economies. The tourism industry and service standards serve as crucial safeguard conditions for the development of tourism economies, while industrial structure acts as a significant driving force. Based on the principles of scientific validity and accessibility, indicators such as tourism resources (Z_1_), economic development level (Z_2_), industrial structure (Z_3_), the number of star hotels (Z_4_), and the number of travel agencies (Z_5_) have been selected. Specifically, the tourism resource indicator is represented by the scores of class-A tourist attractions, the economic development level indicator is represented by the regional GDP, and the industrial structure is represented by the proportion of the third industry’s gross value added to GDP. Given the substantial differences in the attractiveness of class-A tourist attractions at various levels, which result in significant variations in attracting tourists and tourism revenue, it is necessary to assign different weights to A-grade tourist attractions of different levels. Referring to relevant literature [36], the score for class-A tourist attractions (Z1) is calculated as follows: Z1 = 0.25*1A + 0.75*2A + 1.5*3A + 2.5*4A + 5*5A.

### Multiple linear regression perspective

In order to investigate the quantitative patterns of variation between tourism resource conditions, economic development level, tourism industry and service standards, industrial structure, and locational conditions concerning the comprehensive score (F) in the evaluation of tourism economic development, a multiple linear regression formula is employed to describe the relationships among these variables. Subsequently, this analysis aims to determine the extent to which one or more variables influence the comprehensive score (F) of tourism economic development. The formula is as follows:

$$\text{F}={\text{B}}_{0}+{\text{B}}_{1}{\text{Z}}_{1}+{\text{B}}_{2}{\text{Z}}_{2}+\ldots \ldots +{\text{B}}_{\text{K}}{\text{Z}}_{\text{K}}$$ (5)

In the formula, B_0_ is the regression constant; B_1_, B_2_,……, B_K_ are regression coefficients; Z_1_, Z_2_,……, Z_K_ are the independent variables, and the factors with correlation coefficients greater than or equal to 0.8 are selected here, which are the score of class-A tourist attractions(Z_1_), regional GDP(Z_2_), the proportion of tertiary industry(Z_3_), number of star hotels(Z_4_) and number of travel agencies(Z_5_); Y is the dependent variable, which refers to the comprehensive score F of the evaluation of tourism economic development level. And the regression effect was highly significant in the ANOVA (Table 6).

**Table 6:** Linear model coefficient.

Model	Unstandardized coefficient		Standard coefficient	t	Sig.
	B	Standard error			
(constant)	-1.489	.374	-	-3.980	.001
Z_1_	.011	.003	.334	3.482	.003
Z_2_	7.958E-5	.000	.238	1.903	.074
Z_3_	.026	.013	.198	1.969	.066
Z_4_	.008	.002	.385	3.692	.002
Z_5_	-.001	.001	-.126	-1.009	.327

Column B in Table 6 shows the unstandardized coefficients of the regression equation, where the constant line corresponds to the constant term in the model, Z_1_ corresponds to the coefficient of the independent variable class-A tourist attractions score in the model, Z_2_ corresponds to the coefficient of the independent variable regional GDP in the model,……, and so on. Thus, the corresponding model can be obtained as:

$$\text{F}=-1.489\;+\;0.011{\text{Z}}_{1}+7.958{\text{Z}}_{2}+\;0.026{\text{Z}}_{3}+\;0.008{\text{Z}}_{4}-0.001{\text{Z}}_{5}$$ (6)

The t-statistic corresponding to lines Z_1_ and Z_4_ in Table 6 is 3.482 and 3.692, respectively, and the accompanying probability of the t-statistic Sig. is 0.003 and 0.002, respectively, which are much lower than the system default significance level of 0.05, which indicates that the independent variables Z_1_ and Z_4_ play a very significant role in the above multiple linear regression model, while the t-statistic corresponding to lines Z_2_, Z_3,_ and Z_5_ is 1.903, 1.969 and -1.009, respectively, and the companion probability Sig. of the t-statistic was 0.074, 0.066 and 0.327, respectively, which were much larger than the system’s default significance level of 0.05, which indicated that the independent variables Z_2_, Z_3,_ and Z_5_ played a less significant role in the above multiple linear regression model. Therefore, we conclude that the most important factors affecting the difference in tourism economic development of the three major city clusters along the middle reaches of the Yangtze River are class-A tourist attractions (Z_1_) and star hotels (Z_4_), that is, tourism resource conditions and tourism industry and service level.

### Geodetector analysis perspective

**Fig 12:**
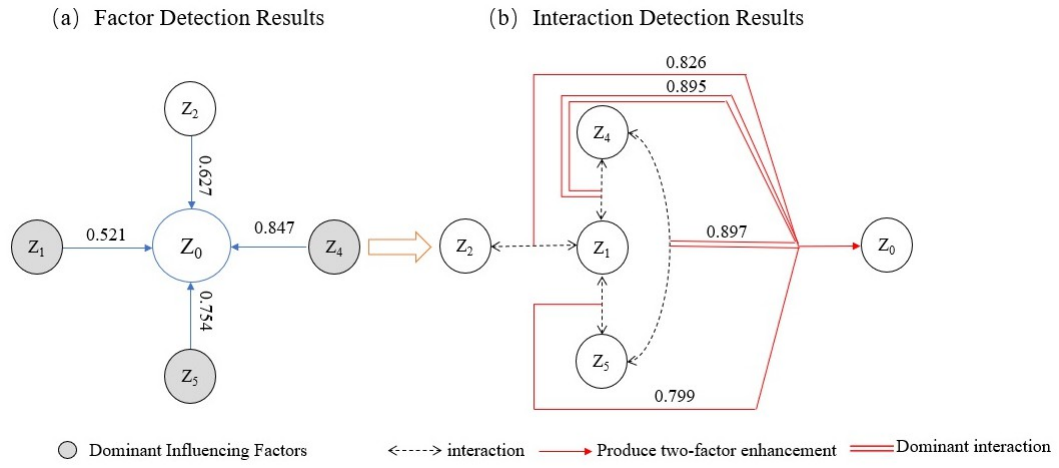
Geographic detection of factors influencing tourism economic disparities. Here are the factor detection and interaction detection for the three major city clusters along the middle reaches of the Yangtze River. Fig 12a shows the results of the factor detection, combining the influencing factors and q-values, with three dominant factors, which are marked in gray; Fig 12b shows the results of the interaction detection.

This passage discusses the selection of various indicators, including total tourism revenue (Z_0_), class-A tourist attractions scores (Z_1_), regional GDP (Z_2_), the proportion of the tertiary industry (Z_3_), the number of star hotels (Z_4_), and the number of travel agencies (Z_5_), to quantitatively analyze the factors influencing differences in tourism economic development. The study focuses on the tourism economic data and related indicators of 23 cities in the middle reaches of the Yangtze River in 2019. By using geodetector and dividing each indicator into five levels through the natural breakpoint method, the study delves into the influencing factors of spatial differences in tourism economic development. The results (Table 7) indicate that three independent variables (Z_1_, Z_4_, Z_5_) are all significant at the 0.05% level, and one independent variable (Z_2_) is significant at the 0.1% level. This suggests that the Z_1_, Z_4_, Z_5_ indicators are important factors affecting spatial differences in tourism economic development, while Z_2_ is a secondary factor. Specifically, class-A tourist attractions, regional GDP, star hotels, and travel agencies all have q-values greater than 0.5. This indicates that class-A tourist attractions, regional GDP, star hotels, and travel agencies are the main common factors influencing the spatial distribution of tourism economic development. In summary, A-grade tourist attractions, star hotels, and travel agencies are identified as the primary factors influencing spatial differences in tourism economic development, while regional GDP is considered a secondary factor (Fig 12a).

**Table 7:** The geodetector results of factors influencing the spatial distribution of tourism economic development.

Detection Indicator	Z_1_	Z_2_	Z_3_	Z_4_	Z_5_
q-value	0.521	0.627	0.446	0.847	0.754
p-value	0.030	0.078	0.131	0.000	0.009

Due to the mutual influence among various indicators, a further analysis of the impact of each factor was conducted through geodetector with a focus on two-factor interactions. The results are illustrated in Fig 12b, revealing that there is a double-factor enhancement interaction among the factors. No independent or weakening relationships were observed. The interaction of all detection factors enhanced the explanatory power for differences in tourism economic development, indicating that the formation of regional differentiation patterns in tourism economic development differences is the result of the joint action of various factors. It is noteworthy that class-A tourist attractions (Z_1_) and the number of star hotels (Z_4_), as well as the number of star hotels (Z_4_) and the number of travel agencies (Z_5_), exhibit dominant interactive effects. This once again emphasizes that class-A tourist attractions, as the foundation of tourism resources, and the tourism industry and service standards based on star hotels and travel agencies are the dominant factors influencing the differences in tourism economic development in the three major city clusters along the middle reaches of the Yangtze River.

## Conclusion and discussion

### Discussion

Through the analysis of the characteristics of the time evolution of the tourism economy, the spatial differentiation characteristics of the tourism economy, and the influencing factors of tourism economic difference, the tourism economy of the three major city clusters along the middle reaches of the Yangtze River is studied in a more comprehensive way, which has good reference significance for the economic development of the city clusters. In the future, the three major city clusters along the middle reaches of the Yangtze River should take integrated and coordinated development as the goal, optimize the current spatial layout of tourism economy, take Wuhan as the main center of integrated and coordinated development of tourism, Changsha and Nanchang as sub-centers, focus on cultivating the tourism economy of Xianning, Huanggang, Jiujiang, Shangrao, Yueyang, Xiangtan and Hengyang, which are important regional nodes, and strengthen the integration of surrounding cities through their driving role, and realize the closeness of tourism economic ties in core city areas; Optimize the transportation accessibility of the axis cities between the core tourism cities, realize the optimization and integration of tourism resources, strengthen the sharing of tourism resources between each other, and realize the overall upgrading of the region; Break down administrative barriers, get rid of the restrictions of provincial barriers, achieve common development, sharing and win-win, and build a policy of integrated and coordinated development of tourism economy.

Due to the difficulty of obtaining data, the analysis of the tourism economic differences between the three major city clusters along the middle reaches of the Yangtze River is not too long, and a longer period of analysis and research is required to better discover the rules. Because there are no county-level tourism statistics, the analysis of the spatial differences in tourism development of the three major city clusters along the middle reaches of the Yangtze River is somewhat crude. There is still room for improvement in the formation mechanism of tourism development differences in the three major city clusters along the middle reaches of the Yangtze River.

### Conclusion

In terms of temporal evolution, the three major city clusters along the middle reaches of the Yangtze River showed a trend of overall differences and fluctuations within each city group decreasing, and the fluctuations of differences between groups increased. The difference in the domestic tourism economy contributes more than 90% to the overall tourism economy difference and narrowing the difference in tourism economy mainly lies in narrowing the gap between domestic tourism economy incomes in various cities. The primacy degree of tourism cities shows an "inverted U-shaped" trend of increasing first and then decreasing. The difference in the Chang-Zhu-Tan city group decreased the fastest, and the difference is currently the smallest. The difference in the Poyang ecological economic zone is the most stable, and the difference is relatively small. The difference in Wuhan city circle has fallen rapidly, but the difference is currently the largest.

In terms of spatial difference, there are significant spatial differences in the proportion of the tourism economy among the three major city clusters along the middle reaches of the Yangtze River. The total tourism economy of Wuhan, Shangrao, Jiujiang, Changsha, and Nanchang is high, while the tourism economic status of Jingdezhen, Shangrao, Jiujiang, Yingtan, Fuzhou, Nanchang, Xiangtan, and Loudi is high. The comprehensive level of the tourism economy can be divided into five levels: the first level is Wuhan and Changsha; Shangrao, Jiujiang, Nanchang, and Jingdezhen form the second level; The cities located at the third level include Zhuzhou, Yingtan, Xiangtan, Fuzhou, Yueyang, Hengyang, Xianning, and Huangshi; The fourth level includes Changde, Ezhou, Xiaogan, Loudi, and Huanggang; Qianjiang, Xiantao, Yiyang, and Tianmen constitute the fifth level.

In terms of influencing factors, examining from both the perspectives of multiple linear regression and geodetector, it is observed that the differences in tourism economic development among the three major city clusters along the middle reaches of the Yangtze River result from the collective impact of various factors. These factors include tourism resource conditions, economic development level, tourism industry and service standards, industrial structure, and locational conditions. The most significant influencing factors are identified as tourism resource conditions based on class-A tourist attractions and the tourism industry and service standards based on star hotels and travel agencies. Other factors also play a certain role in shaping the differences in tourism economic development.
